# *Campylobacter jejuni* infection impacts host-derived miRNAs targeting bacterial and host genes

**DOI:** 10.1128/spectrum.03184-25

**Published:** 2026-01-22

**Authors:** Siddhi Chitre, Raad Z. Gharaibeh, Rachel C. Newsome, Jinmai Jiang, Andrew Brock, Thomas D. Schmittgen, Christian Jobin

**Affiliations:** 1 Department of Medicine, University of Florida College of Medicine 3463, Gainesville, Florida, USA; 2 Department of Molecular Genetics and Microbiology, University of Florida 3463, Gainesville, Florida, USA; 3 Department of Pharmaceutics, College of Pharmacy, University of Florida 3463, Gainesville, Florida, USA; 4 Department of Infectious Diseases and Immunology, University of Florida College of Medicine 12233, Gainesville, Florida, USA; 5 Department of Anatomy and Cell Biology, University of Florida College of Medicine 12233, Gainesville, Florida, USA; Cleveland Clinic Lerner Research Institute, Cleveland, Ohio, USA

**Keywords:** inflammation, virulence, colibactin, cytolethal distending toxin, bacterial consortium, microbiome, miRNA

## Abstract

The intestinal microbiota has been shown to influence host-derived microRNAs (miRNAs), small regulatory RNAs involved in host-pathogen interactions. However, the specific role of individual bacterial species in modulating host extracellular vesicles (EV)-derived miRNA profiles remains unclear. We compared stool miRNA profiles from three groups: (i) Germ-free (GF) mice, (ii) mice colonized with a consortium of 13 bacteria (C13), and (iii) mice colonized with C13 and *Campylobacter jejuni* 81–176 (C13 + *C. jejuni*). miRNA profiling revealed unique differential expression of 8 miRNAs in C13 vs GF, 38 in C13 + *C. jejuni* vs C13, and 159 in C13 + *C. jejuni* vs GF (*P*_adj_ < 0.05), indicating a substantial shift in host miRNA landscape following *C. jejuni* exposure. Notably, C13 + *C. jejuni* mice showed increased expression of miRNAs predicted to target bacterial virulence genes, suggesting a potential host response mechanism aimed at regulating microbial activity. Integration with spatial transcriptomics of the mouse colon revealed that specific EV-derived miRNAs, including mmu-miR-155-5p, mmu-miR-122-5p, mmu-miR-21a-5p, mmu-let-7i-5p, and mmu-miR-142a-3p target epithelial genes involved in immune regulation, inflammation, and barrier function. *C. jejuni* exposure selectively enriched these host miRNAs, suggesting a mechanism by which EVs modulate epithelial transcriptional responses and potentially influence microbial gene expression. In conclusion, *C. jejuni* infection modified host EV-derived miRNA expression profile that may have a potential to bind to the bacterial gene targets.

**IMPORTANCE:**

Host-derived microRNAs (miRNAs) are known to regulate bacterial gene expression and maintain gut homeostasis. However, how these miRNAs survive harsh gut conditions to remain functional is not fully understood. This study tested whether extracellular vesicles (EV) carry microRNAs in the gut and whether infection with the enteric pathogen *Campylobacter jejuni* alters the microRNA profile packaged in these vesicles. We utilized fecal samples from mice, either maintained germ-free (absence of microbiota) or, C13 (defined 13 bacterial consortium), and C13 + *C. jejuni* to analyze the EV-derived miRNA pattern across the groups. Our results revealed distinct sets of miRNAs in each group and suggested possible interactions between these miRNAs and gene transcripts from both the host and bacteria. These findings provide new insights into how *C. jejuni* infection may change communication between the host and its microbiome, potentially affecting gut health and disease.

## INTRODUCTION

A regulated interaction is essential between the host and the gut microbiota to maintain gut homeostasis and disruption of this interaction leading to microbial dysbiosis can cause various gastrointestinal diseases (1). This homeostatic host-microbiota communication is influenced by numerous extrinsic factors such as diet (2), stress (3), antibiotic exposure (4), and pathogenic infection (1) and host-derived factors such as mucus (5), immunoglobulin A (6), anti-microbial peptides (7), and recently miRNA (8, 9). miRNA-mediated communication has been shown to play a critical role in maintaining healthy gut homeostasis (10), but their dysregulation is also associated with inflammatory bowel disease (IBD) (11). Interestingly, bacterial pathogens can affect the expression of the host miRNAs (12). A previous study showed that host-derived miRNAs (mmu-miR-515-5p and mmu-miR-1226-5p) promoted the growth of *F. nucleatum* and *E. coli*, respectively (13). Interestingly, the profile of EV-containing miRNA was altered in the feces of rats exposed to the inflammatory compound dextran sodium sulfate (DSS) compared to healthy rats (14). The authors also demonstrated that treatment with protective miRNAs (e.g., miR-200b-3p) alters the gut microbiome, increasing the abundance of specific beneficial bacteria such as *Lactobacillus* and reducing the abundance of potentially harmful bacteria like *Escherichia* (14). We have previously established that mucosal-associated bacterial communities obtained from biofilm-positive or negative colorectal cancer (CRC) patients differentially affect the production of host-derived miRNA and CRC development in a preclinical model (15). However, our understanding of the role of specific bacteria in influencing miRNA production *in vivo* and subsequent gene targets is limited.

Campylobacteriosis caused by *Campylobacter jejuni (C. jejuni),* although categorized as a self-limiting disease, is a significant cause of concern due to the presence of various virulent factors (16, 17). Studies have indicated the enrichment of *Campylobacter* species in IBD (18) and within tumor and feces of CRC patients (19–22). Recent studies have also demonstrated the presence of *C. jejuni* virulence genes *cdt*A, B, and C in diarrheal patients (23, 24). We have previously demonstrated that *C. jejuni-*derived CDT has tumorigenic and metastatic properties in pre-clinical models (25, 26). Since campylobacteriosis is one of the most prevalent forms of bacteria-induced gastroenteritis, it is important to understand the detailed host responses to this pathogen. We previously established a consortium of 13 bacteria (C13) representing the dominant bacterial phyla (27) in the mouse gut as a tractable system to study host response to *Campylobacter jejuni* 81–176 infection in GF *Il10^−/^*^−^ mice. We observed that *C. jejuni* infection of C13-colonized mice triggered host inflammatory gene expression and increased *E. coli* colonization in the distal colons compared to C13 alone (27). In this study, we demonstrated that the extracellular vesicle (EV)-derived miRNA profile is altered by *C. jejuni* infection*,* which is associated with the production of miRNAs predicted to bind bacterial virulence gene targets. We also leveraged EV miRNA profiling and integrated these data with spatial transcriptomics to examine how *C. jejuni* infection modulates host EV-derived miRNAs and their targeting of gut epithelial genes, providing insights into host-microbiota communication and pathogen-mediated shifts in microbial and epithelial dynamics.

## RESULTS

### Stool-derived extracellular vesicles retain size and structural integrity and are free from bacterial RNA

**Fig 1:**
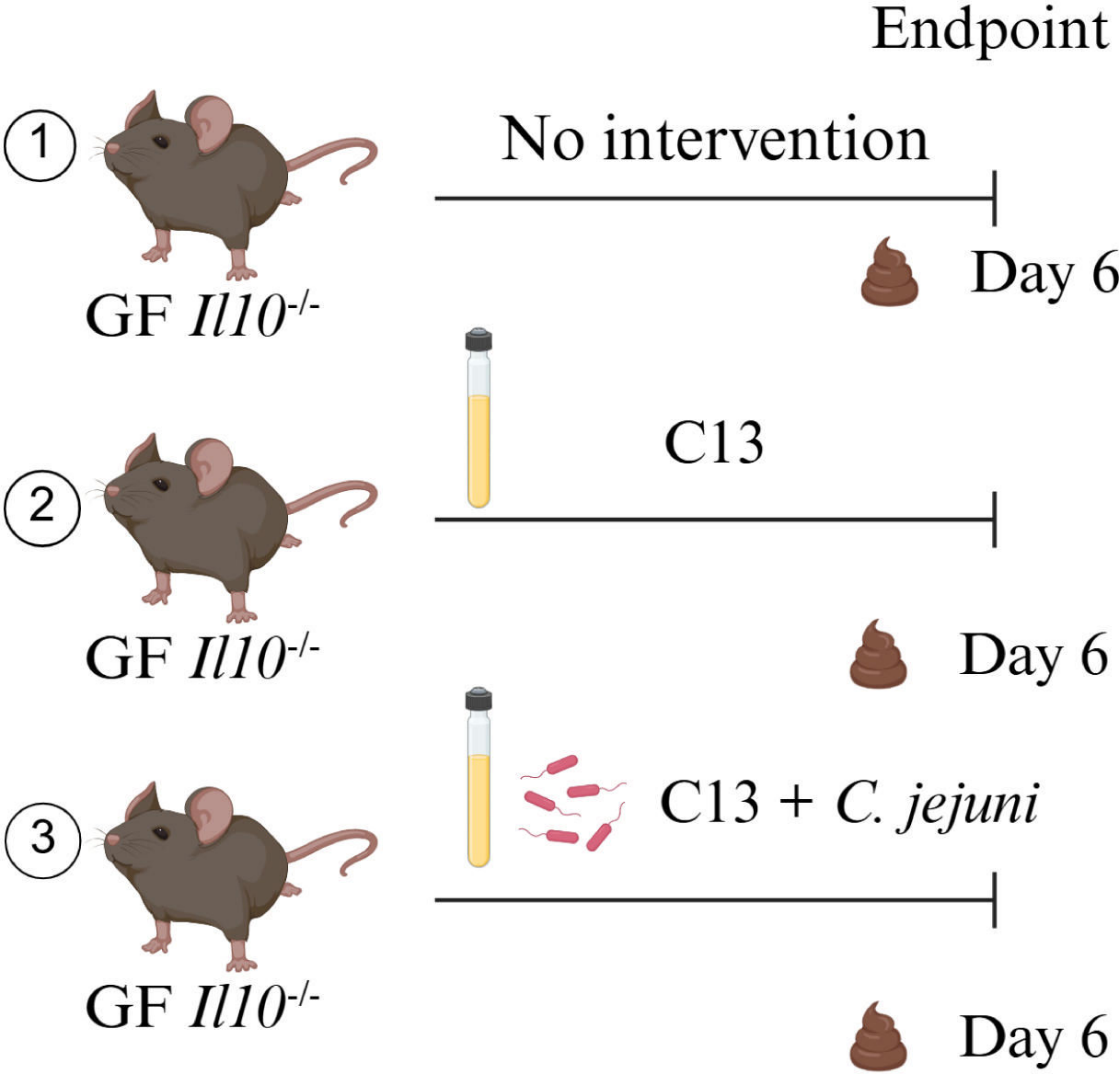
Experimental workflow for investigating host-bacterial communication in GF, C13, and C13 + *C. jejuni* infection.

**Fig 2:**
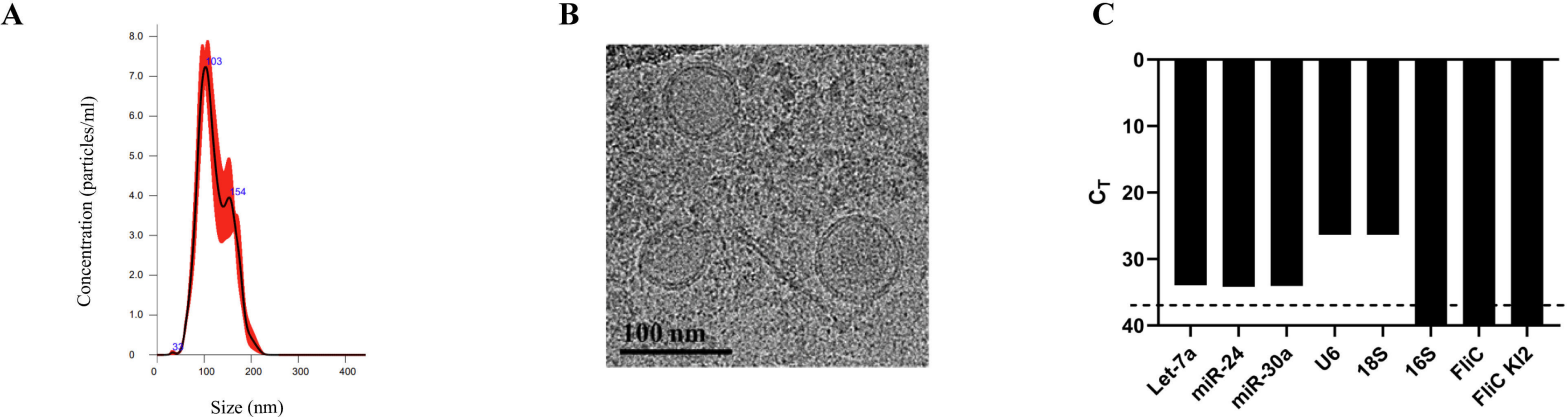
EV-derived miRNA demonstrates a differential miRNA profile. (**A**) The particle size distribution and concentration of EVs were assessed using NTA. (**B**) Cryo-TEM images of mouse stool EVs isolated scale bar size = 100 nm. (**C**) Gene expression of mammalian and prokaryotic gene transcripts in stool-derived EVs. The dashed line indicates the limit of detection for the RT-qPCR.

We previously found that biofilm-positive or negative bacteria differentially affect the production of host-derived miRNA in a CRC preclinical model (15). The establishment of a bacterial consortium sensitive to the presence of *C. jejuni* (27) represents a unique opportunity to interrogate the impact of bacteria on host miRNA expression. The inflammation status of the three groups, GF, C13 colonized, and C13 + *C. jejuni*-infected mice, has been characterized previously (27) and showed minimal inflammation in GF (mean inflammation score 0.8) and C13-colonized mice (mean inflammation score 1.5), and pronounced mucosal damage, goblet cell loss, and immune cell infiltration in C13 + *C. jejuni-*infected mice (mean inflammation score 4). Using this system, we sought to understand the impact of this pathogen on host miRNA. To investigate this question, we isolated and characterized the EVs purified from the feces of GF, C13, and C13 + *C. jejuni*-colonized mice (Fig. 1). EVs were isolated by ultracentrifugation and subjected to the nanoparticle tracking analysis system. This analysis detected particles ranging from 33 to 200 nm, with an average of 121.6 nm in size, with two detected peaks at 103 nm and 154 nm, compatible with EV phenotype (Fig. 2A). The cryo-TEM microscopy platform revealed a classic cup shape morphology with a lipid bilayer demonstrating the presence of EVs in the feces of these mice (Fig. 2B). To confirm that the stool EV isolations are free of bacteria and bacterial outer membrane vesicles (OMVs), we used RT-qPCR to analyze the stool EV RNA for eukaryotic and prokaryotic transcripts. The expression levels of these markers confirmed that our EV purification was free from any prokaryotic RNA contamination, as demonstrated by Ct values below the limit of detection for bacterial genes and positive for host eukaryotic markers (Fig. 2C).

### Host-derived miRNA profile is sensitive to bacterial composition

**Fig 3:**
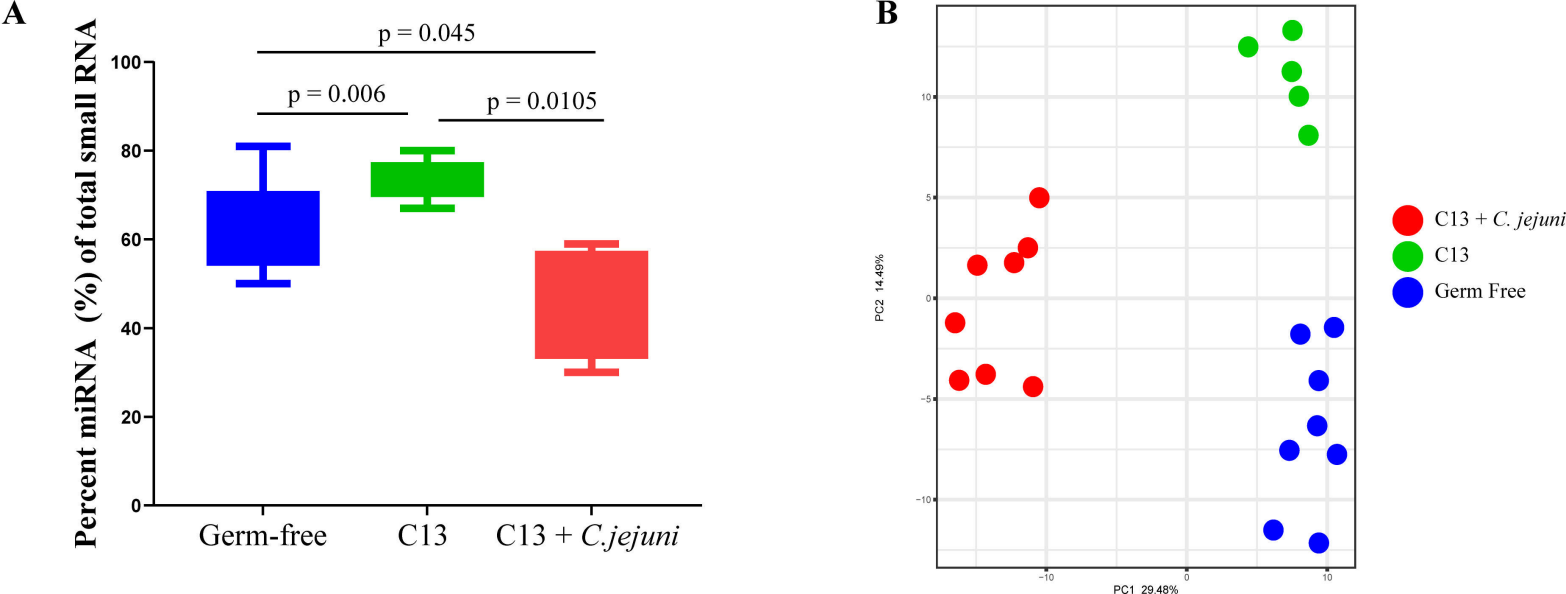
The presence of *C. jejuni* in C13 altered miRNA profile in mice. (**A**) Quantification of miRNA isolated from fecal EVs across all groups GF (*n* = 9), C13 (*n* = 6), C13 + *C. jejuni* (*n* = 8). *P*_adj_-values from R’s two-sample Wilcoxon test after multiple testing correction. (**B**) PCA plot showing sample clustering based on their miRNA profiles in GF (*n* = 8), C13 (*n* = 5), and C13 + *C. jejuni* (*n* = 8) groups. Differences in sample clustering were tested using R’s gls: C13 vs GF *P*_adj_ value = 2.48 × 10^−6^, C13 + *C. jejuni* vs GF *P*_adj_ value = 1.71 × 10^−12^, and C13 + *C. jejuni* vs C13 *P*_adj_ value = 1.36 × 10^−8^. Statistical significance was defined as *P* < 0.05.

To study the impact of *C. jejuni* infection on host EV-derived miRNA profile, we extracted and sequenced miRNA from purified EVs from the feces of GF, C13, and C13 + *C. jejuni*-colonized mice. We observed that *C. jejuni* modulates the miRNA profile of mouse stool EVs. Of the total small RNA from EVs, the C13 + *C. jejuni* feces contained 46% miRNA, whereas EV from the feces of GF and C13 mice had an average of 64% and 74% miRNA, respectively (Fig. 3A; Table S1: https://figshare.com/s/824c92cec2bd7b18bd4e). Principal Component Analysis (PCA) of remaining miRNAs showed three distinct clusters corresponding to the three groups (GF, C13, and C13 + *C. jejuni*) (Fig. 3B), suggesting that the presence of specific bacteria influences miRNA expression profile.

### *C. jejuni* infection induces unique host EV miRNA expression patterns

**Fig 4:**
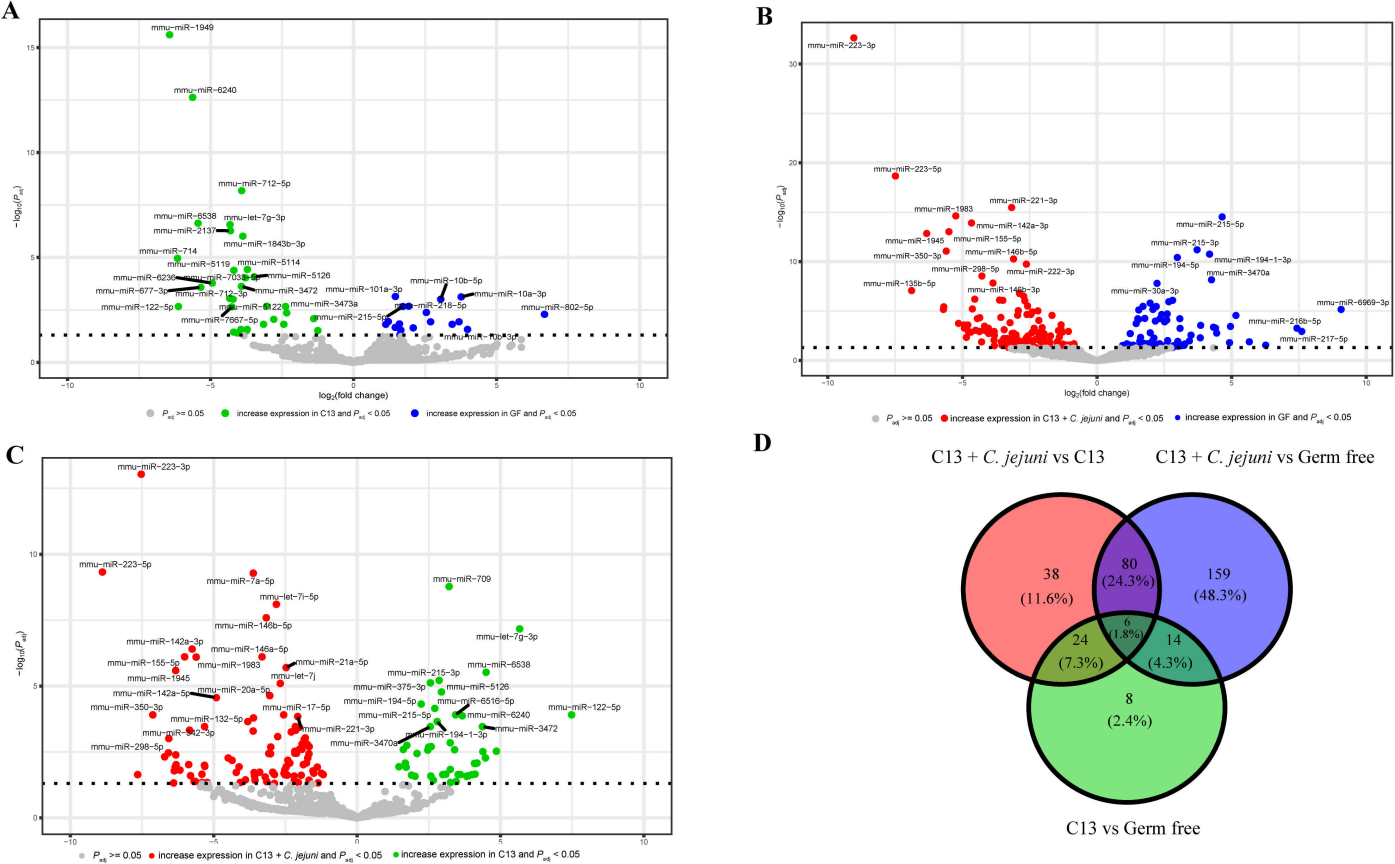
Distinct miRNA signatures observed in GF (*n* = 8), C13 (*n* = 5), and C13 + *C. jejuni* (*n* = 8) infected mice. (**A**) Volcano plot of differentially expressed miRNAs in C13 vs GF. Horizontal dotted line showing −log_10_(0.05). (**B**) Volcano plot of differentially expressed miRNAs in C13 + *C. jejuni* vs GF. Horizontal dotted line showing −log_10_(0.05). (**C**) Volcano plot of differentially expressed miRNAs in C13 + *C. jejuni* vs C13. Horizontal dotted line showing −log_10_(0.05). (**D**) Venn diagram representing shared and unique miRNAs that are significantly and differentially expressed in the three experimental groups. Statistical significance was defined as *P* < 0.05.

To further define differential miRNA responses in this cohort, we analyzed expression patterns across GF, C13, and C13 + *C. jejuni* groups. We observed that C13 colonization significantly induces the expression of different miRNAs as compared to GF (Fig. 4A). Similarly, C13 + *C. jejuni* induces the expression of different miRNAs as compared to GF (Fig. 4B). Furthermore, the addition of *C. jejuni* to the C13 strongly modifies the miRNA expression (Fig. 4C). To further characterize the significantly differentially expressed miRNAs between the three groups, we compared the overlapping miRNA between the three comparisons and identified 8 miRNAs that are unique to the C13 vs GF comparison, 38 unique to C13 + *C. jejuni* vs C13 comparison, and 159 unique to C13 + *C. jejuni* vs GF comparison (Fig. 4D). This suggests that the presence of *C. jejuni* altered both the number and the identity of the significant and differentially expressed miRNAs compared to GF and C13 alone.

### Relationship between EV-derived miRNA with mammalian and bacterial gene target

miRNA sequencing revealed differentially expressed host miRNAs across all three conditions (Table S2: https://figshare.com/s/824c92cec2bd7b18bd4e). Using Probability of Interaction by Target Accessibility (PITA), we predicted several host and bacterial binding partners for these miRNAs. To evaluate possible regulatory effects, we compared the predicted targets with gene expression profiles from a previously generated fecal RNA-seq data set from the same experimental study (27). Although multiple miRNAs were differentially expressed, we highlight four miRNAs that were significantly upregulated and downregulated in the C13 + *C. jejuni* colonized mice and analyzed their predicted mouse gene transcript targets (Table 1, Table S3: https://figshare.com/s/824c92cec2bd7b18bd4e).

**TABLE 1:** miRNAs differentially altered in C13 + *C. jejuni* with their predicted gene targets^*a*^^,^*^b^*

miRNA significant and upregulated in C13 + *C. jejuni* (this study)	Predicted mouse gene target expression levels from RNA-seq data	Expression of mouse gene targets in C13 + *C. jejuni*	Mouse gene function
↑ mmu-miR-223-3p	↓ *Sdc2* (Syndecan 2) *	Decreased	Cell differentiation
↑ mmu-miR-223-5p	↓ *Lin7a* (lin-7 homolog A, crumbs cell polarity complex component)	Decreased	Cell polarity
↑ mmu-miR-7a-5p	↓ *Sike1*(suppressor of IKBKE 1) *	Decreased	Inflammation
↑ mmu-let-7i-5p	↓ *Gab1* (growth factor receptor bound protein 2-associated protein 1)	Decreased	Inflammation

miRNA significant and downregulated in C13 + *C. jejuni* (this study)	Predicted mouse gene target expression levels from RNA-seq data	Expression of mouse gene targets in C13 + *C. jejuni*	Mouse gene function
↓ mmu-miR-709	↑ *Myc* (myelocytomatosis oncogene)	Increased	Proto-oncogene
↓ mmu-miR-6538	↑ *Cdkn1a* (cyclin dependent kinase inhibitor 1A)	Increased	Cell cycle
↓ mmu-miR-215-3p	↑ *Il2ra* (interleukin 2 receptor, alpha chain)	Increased	Immune tolerance
↓ mmu-miR-375-3p	↑ *Jak2* (Janus kinase 2)	Increased	Signal transduction

^*a*^ Notably, among the experimentally validated gene targets, some of the predicted miRNA-target interactions were identified as weak (indicated with asterisk ‘*’ in the table).

^*b*^ The symbols “↑” and “↓” indicate upregulated and downregulated expression, respectively.

We then investigated the presence of any EV-derived miRNA that can regulate bacterial genes. Due to the large data sets produced by PITA analysis, we focused on two miRNAs with the highest expression in each group that were either upregulated or downregulated in C13 + *C. jejuni* or C13 (Table S4: https://figshare.com/s/824c92cec2bd7b18bd4e). The miRNA mmu-miR-223-3p was significantly overexpressed in C13 + *C. jejuni*-infected mice. PITA analysis predicted that this miRNA could bind several bacterial genes. Examination of these bacterial genes in our previously published RNA-seq data set revealed that their expression of bacterial genes corresponding to nutrient transport, iron uptake, metabolism, and cell wall biosynthesis was decreased in C13 + *C. jejuni* compared to C13, suggesting a potential repressive effect of mmu-miR-223-3p on these targets. Interestingly, we also observed an increase in the bacterial virulence associated with gene transcripts including iron uptake and chemotaxis in *E. coli* (Table 2).

**TABLE 2:** mmu-miR-223-3p significant and overexpressed in C13 + *C. jejuni*

Binding score	Increased bacterial gene expression in C13	Bacterial species
−20.37	SusC/RagA family TonB-linked outer membrane protein	*Bacteroides acidifaciens*
−17.98	TonB-dependent receptor	*Bacteroides caecimuris*
−17.45	YncE family protein	*Parabacteroides distasonis*
−16.08	ATP-binding protein	*Parabacteroides goldsteinii*
−14.63	UDP-glucose 4-epimerase GalE	*Blautia caecimuris*
−13.51	MraY family glycosyltransferase	*Ligilactobacillus murinus*

Binding score	Increased bacterial gene expression in C13 + *C. jejuni*	Bacterial species
−16.66	Lysine exporter LysO family protein	*Parabacteroides goldsteinii*
−15.92	Iron uptake system protein EfeO	*Escherichia coli*
−15.67	Flavodoxin domain-containing protein	*Paraclostridium bifermentans*
−14.72	UTP-glucose-1-phosphate uridylyltransferase	*Akkermansia muciniphila*
−14.63	Chemotaxis protein CheA	*Escherichia coli*
−13.07	DedA family protein	*Campylobacter jejuni*

Conversely, mmu-miR-709, which was downregulated in C13 + *C. jejuni*-infected mice, was predicted by PITA to target several bacterial gene transcripts. Examination of these transcripts in our previously published RNA-seq data set showed increased expression of genes involved in nutrient uptake, amino acid metabolism, DNA binding and repair, and energy metabolism across the C13 consortium. In *C. jejuni*, transcripts encoding flagellar proteins were also elevated, suggesting enhanced motility in response to infection (Table 3).

**TABLE 3:** mmu-miR-709 significant and downregulated in C13 + *C. jejuni*

Binding score	Increased bacterial gene expression in C13	Bacterial species
−23.68	RagB/SusD family nutrient uptake outer membrane protein	*Bacteroides caecimuris*
−21.98	Aspartate aminotransferase family protein	*Ligilactobacillus murinus*
−21.37	MATE family efflux transporter	*Bacteroides acidifaciens*
−19.78	Protein arginine kinase	*Staphylococcus xylosus*
−19.7	ATP-binding protein	*Blautia caecimuris*
−19.23	Helix-hairpin-helix domain-containing protein	*Parabacteroides distasonis*
−19.23	ISL3 family transposase	*Ligilactobacillus murinus*
−16.14	Dihydrolipoamide dehydrogenase	*Enterococcus hirae*

Binding score	Increased bacterial gene expression in C13 + *C. jejuni*	Bacterial species
−24.81	Glycoside hydrolase family 30 protein	*Parabacteroides goldsteinii*
−23.37	3-Deoxy-7-phosphoheptulonate synthase	*Akkermansia muciniphila*
−20.02	Flagellar protein export ATPase FliI	*Campylobacter jejuni*
−19.69	Carbamoyl-phosphate synthase large subunit	*Escherichia coli*
−15.57	Copper-translocating P-type ATPase CopA	*Enterococcus hirae*

We observed that in C13, mmu-miR-6240 is associated with an increased expression of bacterial gene transcripts involved in nutrient uptake, signal transduction, and transport, which maintain bacterial homeostasis. Following C13 + *C. jejuni* infection, mmu-miR-6240 was downregulated, which may have resulted in increased expression of gene transcripts responsible for metabolism, fatty acid biosynthesis, and amino acid metabolism in C13 + *C. jejuni* (Table 4).

**TABLE 4:** mmu-miR-6240 significant and overexpressed in C13

Binding score	Increased bacterial gene expression in C13	Bacterial species
−23.64	SPFH domain-containing protein	*Blautia caecimuris*
−22.8	Ammonia-dependent NAD (+) synthetase	*Ligilactobacillus murinus*
−22.33	TonB-dependent receptor	*Parabacteroides distasonis*
−22.23	Glycerate kinase	*Enterococcus hirae*
−20.54	DUF2339 domain-containing protein	*Bacteroides acidifaciens*
−17.81	Lactonase family protein	*Enterococcus hirae*

Binding score	Increased bacterial gene expression in C13 + *C. jejuni*	Bacterial species
−23.24	XRE family transcriptional regulator	*Parabacteroides goldsteinii*
−22.95	Enoyl-ACP reductase	*Campylobacter jejuni*
−22	TIGR03749 family integrating conjugative element protein	*Escherichia coli*
−19.12	Aminotransferase class I/II-fold pyridoxal phosphate- dependent enzyme	*Akkermansia muciniphila*
−13.61	Histidine kinase dimerization/phospho-acceptor domain-containing protein	*Paraclostridium bifermentans*

Next, we focused on mmu-miR-10a-3p, which is downregulated in C13, leading to increased expression of bacterial gene transcripts involved in protein synthesis and ATP hydrolysis for substrate transporters across the C13 consortium. With this downregulation, we also observed a significant increase in the bacterial gene expression related to nutrient uptake, increased bacterial adhesion and colonization in the hosts, bacterial motility, adhesion, and DNA replication (Table 5), to name a few biological functions. Collectively, these analyses suggest that *C. jejuni* infection alters the host EV miRNA profile, which, in turn, may influence bacterial gene expression and contribute to shifts in gut microbiota composition.

**TABLE 5:** mmu-miR-10a-3p significant and downregulated in C13

Binding score	Increased bacterial gene expression in C13	Bacterial species
−15.98	50S ribosomal protein L11 methyltransferase	*Bacteroides acidifaciens*
−15.98	50S ribosomal protein L11 methyltransferase	*Bacteroides caecimuris*
−13.54	DUF4352 domain-containing protein	*Enterococcus hirae*
−13.31	ABC transporter ATP-binding protein	*Blautia caecimuris*

Binding score	Increased bacterial gene expression in C13 + *C. jejuni*	Bacterial species
−15.29	RagB/SusD family nutrient uptake outer membrane protein	*Parabacteroides goldsteinii*
−14.47	Uroporphyrinogen decarboxylase	*Campylobacter jejuni*
−13.26	SdrD B-like domain-containing protein	*Paraclostridium bifermentans*
−13.99	Motility associated factor glycosyltransferase family protein	*Campylobacter jejuni*
−13.54	Primosomal protein N'	*Akkermansia muciniphila*
−13.39	Sigma-E factor regulatory protein RseB	*Escherichia coli*

Interestingly, our analysis showed 25 unique miRNAs significantly upregulated in C13 and predicted to target *E. coli* pks genes such as *clbB*, *clbD*, *clbF*, *clbG*, *clbH*, *clbJ*, *clbM*, and *clbQ*. Correspondingly, these genes were all significantly upregulated in the feces of C13 + *C. jejuni* colonized mice (Table S5: https://figshare.com/s/824c92cec2bd7b18bd4e). Interestingly, analysis of *C. jejuni cdt* gene transcripts revealed significant upregulation in the C13 + *C. jejuni* group. In this group, we observed that mmu-miR-28a-3p (predicted to bind to *C. jejuni cdt*A) and mmu-miR-1981-5p (predicted to bind to *C. jejuni cdt*A and *cdt*C) were also significantly upregulated. Additionally, mmu-miR-680, which was significantly upregulated in C13, was predicted to target *cdt*C. These observations suggest that host miRNAs may influence the expression of *C. jejuni* toxin genes, potentially contributing to host protection or, alternatively, modulating bacterial gene expression. Further experimental studies are needed to clarify the functional impact of these miRNAs.

These findings showed that the addition of *C. jejuni* to a defined consortium drastically modified host and bacterial gene expression, which is accompanied by altered expression of miRNAs that are predicted to interact with both mammalian and bacterial genes.

### Spatial transcriptomics reveal stool-derived EV miRNAs targeting key gut epithelial genes

Using spatial transcriptomics data from our previous work (27), we observed that *C. jejuni* colonization induced region-specific transcriptional changes in the colonic tissue depending on the bacterial abundance. The regions analyzed were defined based on the presence or absence of *C. jejuni* in the colon*,* as determined by fluorescence *in situ* hybridization (FISH) staining assay (27). Notably, some regions were relatively depleted of *C. jejuni*, which may reflect local differences in microbial colonization, mucus layer composition, or epithelial susceptibility. To determine whether host EV-derived miRNAs contributed to these changes, we integrated spatial transcriptomics results with stool EV miRNA profiles and focused on experimentally validated miRNA–mRNA interactions (Table S6: https://figshare.com/s/824c92cec2bd7b18bd4e). This analysis revealed several miRNAs with distinct expression patterns, each predicted to target key genes involved in immune activation, epithelial repair, or inflammation. Among them, mmu-miR-155-5p was the most prominent, showing marked upregulation in C13 + *C. jejuni* mice. It was predicted to regulate transcripts including *Saa3*, *Il1a*, *Ido1*, *Lyz2*, and *S100a8*, all genes associated with pro-inflammatory or antimicrobial responses. Consistent with this, regions enriched in *C. jejuni* exhibited strong upregulation of *Saa3*, *Il1a*, *Lyz2*, and *S100a8*, whereas *Ido1*, an immune tolerance gene, was significantly downregulated in *C. jejuni-*depleted tissue regions.

mmu-miR-122-5p, which was upregulated in the C13 group but reduced in C13 + *C. jejuni* mice, was predicted to bind *Cxcl9*, *Krt19*, *Ccl5*, and *Kctd12*. Notably, *Cxcl9, Ccl5*, and *Kctd12* expression was elevated in C13 + *C. jejuni* mice, suggesting that diminished miR-122-5p expression may relieve repression on *Cxcl9*, *Ccl5*, and *Kctd12*, enhancing immune chemokine signaling. Similarly, *Krt19* genes involved in epithelial integrity were downregulated in regions lacking *C. jejuni*, indicating spatially distinct regulatory mechanisms.

In addition, mmu-miR-21a-5p was significantly increased in C13 + *C. jejuni* mice, with its target gene transcript *S100a8* also showing elevated expression in *C. jejuni*-enriched regions, implicating this miRNA–mRNA pair in pro-inflammatory signaling. Similarly, mmu-let-7i-5p and its target *Itgb2* were both upregulated in *C. jejuni*-enriched areas, indicating coordinated regulation of immune cell adhesion pathways during bacterial colonization. mmu-miR-142a-3p was upregulated in C13 + *C. jejuni*, whereas its predicted target *Cystm1* was reduced in *C. jejuni*-depleted tissue regions, suggesting a potential role in modulating epithelial defense mechanisms.

Taken together, these findings demonstrate that *C. jejuni* colonization reshapes the host EV-miRNA landscape in a spatially resolved manner. The enrichment of miRNAs such as miR-155-5p and the loss of regulatory miRNAs like miR-122-5p suggest a coordinated mechanism by which host-derived miRNAs modulate immune and epithelial gene expression in response to *C. jejuni* colonization.

## DISCUSSION

The relationship between host response to enteric pathogens and induction of luminal miRNAs is poorly understood. We previously established a novel tractable system for studying *C. jejuni* host response in the presence of a curated consortium of 13 bacteria (C13) representing four dominant phyla in the murine gut (27). This established consortium provided a foundation for the current investigation, to understand previously unidentified host responses.

In this study, we observed that the C13 + *C. jejuni* group had significantly less miRNA encapsulated in fecal EVs, suggesting that the pathogen may interfere with the biogenesis of EVs and/or miRNA. This finding suggests a potential role for *C. jejuni* in altering the miRNA content within the EVs, as seen with infection with other pathogenic bacteria (12). We observed an upregulation of miRNAs such as mmu-miR-10b-5p and mmu-miR-215-5p in GF mice, which have a role in maintaining gut homeostasis. For example, a global deficiency of mmu-miR-10b-5p leads to alterations in gut functions such as increased intestinal permeability, dysregulation of tight junctions (28), and disruption of gut immune response (29). mmu-miR-215-5p plays a role in the host by suppressing inflammation and by repressing the inflammatory Interleukin Enhancer Binding Factor 3 (ILF3) and LRR binding FLII interacting protein 1 (LRRFIP1) (30). These data suggest that while GF mice express miRNAs involved in gut homeostatic functions, they can be susceptible to infections due to the abundance of miRNAs that contribute to the suppression of inflammation, which is supported by previous reports that GF mice are prone to infectious diseases (31). These data suggest that a healthy gut microbiota (as established by the C13 consortium) leads to the production of specific miRNAs that maintain cellular homeostasis and protect from disease development. However, this symbiotic balance shifts to dysbiosis after the addition of the pathogenic *C. jejuni* to the consortium. We also observed an altered miRNA profile associated with inflammation and cancer metastasis. For example, mmu-miR-221-3p, mmu-let-7i-5p, mmu-miR-142a-3p are upregulated in the C13 + *C. jejuni* group and are associated with inflammation (32–34). Additionally, mmu-miR-223-3p and mmu-miR-7a-5p were increased in this group and are associated with the upregulation of Stat3 and Jak2 expression, respectively. Interestingly, He et al. (25) recently demonstrated that *C. jejuni* CdtB enhanced cancer metastasis through the JAK2/STAT3 signaling pathway, subsequently increasing the expression of pro-metastatic matrix metalloprotease 9 (MMP9). Thus, our data suggest a possible host-derived miRNA link in the mechanism of *C. jejuni* pathogenicity.

We identified miRNA and predicted their potential gene targets across the consortium. For example, mmu-miR-6240, which is significantly increased in C13, is predicted to bind to genes implicated in nutrient uptake, signal transduction, and transport across *Blautia*, *Bacteroides*, *Enterococcus*, *Ligilactobacillus*, and *Parabacteroides* suggesting a regulatory mechanism for maintaining bacterial homeostasis. Following *C. jejuni* inclusion, there is an imbalance in the regulation as seen by significant downregulation of another miRNA, mmu-miR-468-3p, which may lead to increased expression of pathogenic genes such as *E. coli* colibactin ClbH gene.

We also observed a significant increase of mmu-miR-223-3p in the C13 + *C. jejuni* group. This miRNA is predicted to bind to distinct gene transcripts involved in nutrient transport, iron uptake, metabolism, and cell wall biosynthesis, including *Blautia, Bacteroides, Ligilactobacillus,* and *Parabacteroides* suggesting a potential inhibitory mechanism that could cause a shift in gut microbiota composition. Additionally, mmu-miR-223-3p is predicted to bind to bacterial gene transcripts responsible for iron acquisition and chemotaxis across *Akkermansia, Campylobacter, Escherichia,* and *Parabacteroides,* suggesting a potential activation of virulent genes that would confer survival advantages over other bacteria. Interestingly, we observed that the presence of *C. jejuni* in the C13 led to an increased expression of miRNA mmu-miR-1981-5p, predicted to bind *cdt*A and *cdt*C transcripts. Host-derived miRNA targeting *C. jejuni* CDT subunits may represent a mechanism to counteract the pathogenic effects of CDT.

Integrating previously generated spatial transcriptomics data with host EV-derived miRNA profiles highlights a novel layer of host-microbiota communication. Spatial transcriptomics allowed us to identify key epithelial genes whose expression patterns potentially may be modulated by EV miRNAs, including mmu-miR-155-5p, mmu-miR-122-5p, mmu-miR-21a-5p, mmu-let-7i-5p, and mmu-miR-142a-3p. These miRNAs target genes involved in pro-inflammatory signaling, antimicrobial defense, chemokine-mediated immune recruitment, epithelial repair and barrier maintenance, immune cell adhesion, and immune tolerance, suggesting that host EVs can fine-tune epithelial responses to microbial colonization. Together, integrating spatial and host EV-derived miRNA data offers a powerful strategy to dissect host-microbiota crosstalk. We previously demonstrated that *C. jejuni* infection in GF *Il10^−^/*^−^ mice leads to rapid disruption of mucosal architecture, characterized by the loss of epithelial and goblet cells and marked immune cell infiltration by day 14 post-infection (35). In a separate study (36), acute ulcerative colitis in *Il10^−^/*^−^ mice was characterized by colonic ulceration, bleeding, diffuse mucosal and submucosal infiltrates, and loss of goblet cells and crypts by day seven post-infection. Together, these findings indicate that infection triggers both direct epithelial damage and secondary effects associated with inflammation and tissue remodeling. In this context, the EV changes observed in our study are likely to arise from multiple cellular sources. As our GF model does not allow precise cell-type attribution, it remains unclear whether epithelial or immune cells contribute most significantly to the EV pool. Future studies employing cell-type-specific EV isolation or labeling strategies to better define the origins and functional roles of EVs during *C. jejuni* infection. Nevertheless, our study provides the first characterization of EV-derived miRNAs in the context of *C. jejuni* infection. Future studies investigating the profile of EV-derived miRNA in *C. jejuni* mono-associated GF mice may provide additional information on the contribution of this pathogenic bacterium to host-microbe interaction.

Further investigation is also warranted to understand the functional implications of miRNA binding in homeostasis and pathogenic states. The differential profile of miRNAs generated by the host upon colonization status (GF, C13, and C13 + *C. jejuni*) highlights their potential significance in regulating host-microbe interaction. The main limitation of this study is the correlative nature of the complex miRNA-bacterial interactions presented in the paper. The data are based on *in silico* analysis, and functional experiments will be needed to demonstrate cause-effect relationships with candidate miRNAs. The synthesis of candidate miRNA and target delivery in bacterial cultures of individual C13 members may allow the assessment of bacterial gene expression in a controlled manner.

### Conclusion

**Fig 5:**
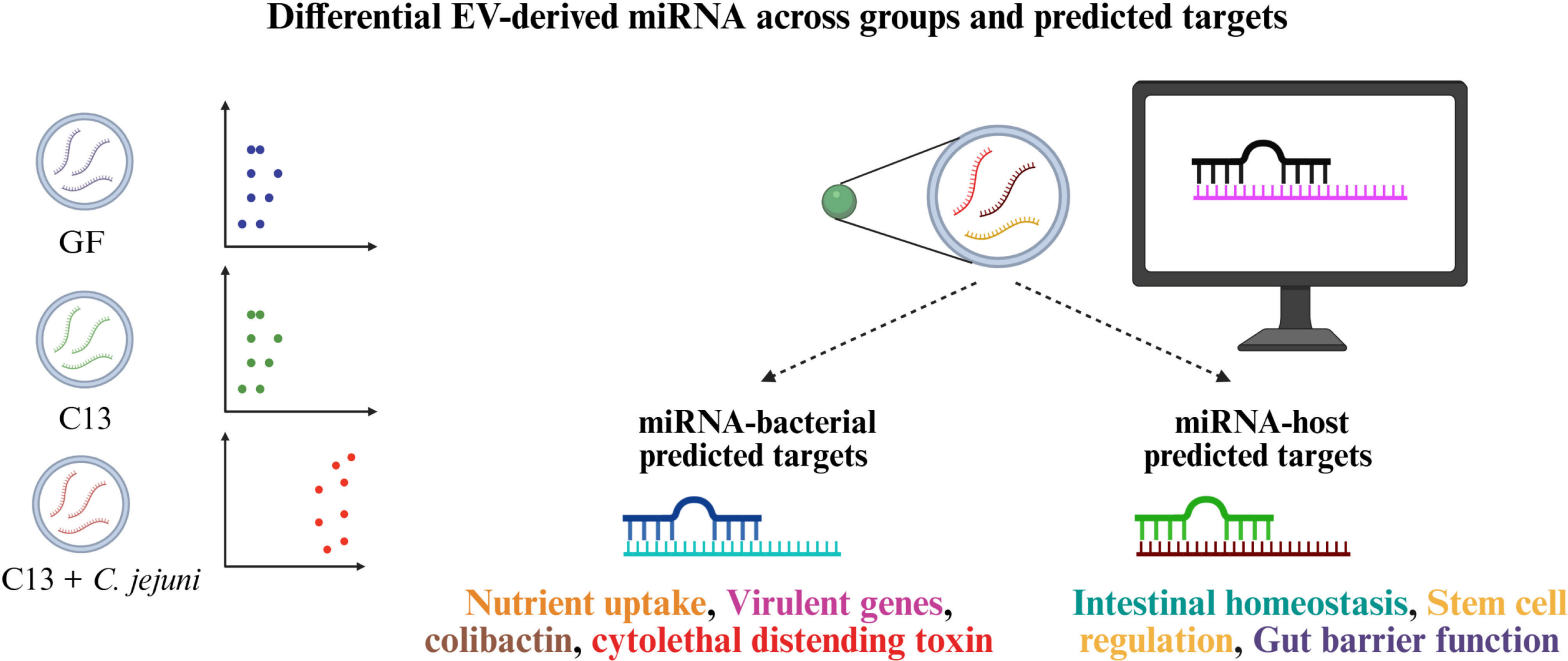
Graphical representation of significant findings. Fecal EV-derived miRNA sequencing from GF, C13, and C13 + *C. jejuni* group revealed distinct miRNA clustering. Predicted interactions between miRNAs and host or bacterial gene transcripts were identified using PITA analysis. These results highlight potential targets for further functional validation.

In conclusion, we demonstrate that *C. jejuni* infection alters fecal EV-derived miRNA profile (Fig. 5). The miRNAs abundantly expressed upon *C. jejuni* infection are predicted to bind to the bacterial virulence genes thereby modulating their function.

## MATERIALS AND METHODS

### Mice experiment

All animal experiments were approved by the Institutional Animal Care and Use Committee (IACUC) at the University of Florida (UF) and performed at UF Animal Care Facilities (IACUC Protocol #202200000637). Mice fecal samples used in this study are a part of an experimental cohort previously described in a separate manuscript (27) from which stool samples were collected and analyzed here. Briefly, 6–10 week old, mixed gender GF *Il10*^−/−^ (129/SvEv) mice were divided into three groups: (i) GF, (ii) C13 consortium containing the four most prominent phyla present in the healthy mouse gut (C13), and (iii) C13 containing *C. jejuni* 81–176 (C13 + *C. jejuni*). Mice were gavaged with the different bacterial consortium (10^6^ CFU). C13 + *C. jejuni* colonized mice were monitored daily for the clinical signs of campylobacterosis, allowing us to capture EV-miRNA and transcriptional changes associated with active infection while minimizing severe pathology. Stool samples were collected on day 6 post-gavage for all the groups.

### Isolation of EVs from stool samples of GF, C13, and C13 + *C. jejuni* infected mice

Fecal samples from GF, C13, and C13 + *C. jejuni*, snap-frozen in liquid nitrogen and stored in −80°C were utilized for this analysis. Due to limited stool quantity obtained from one mouse in C13 + *C. jejuni* group, we pooled this sample with the stool sample from other mouse within the same group to ensure sufficient EV yields, resulting in a final sample size of GF (*n* = 9), C13 (*n* = 6), and C13 + *C. jejuni* (*n* = 8). EVs were isolated via standard differential centrifugation and ultracentrifugation methodologies following an established mouse stool EV isolation protocol (37) but with some modifications (38). Briefly, flash frozen (stored in −80°C) mouse stool from individual mice was thawed on ice, and individual stool pellets were placed in 50 mL conical tubes with 15 mL protease inhibitor cocktail solution (one tablet per 10 mLs ice cold PBS) and incubated at 4°C for 30 min. After 30 min of soaking at 4°C, the tube was vortexed at maximum speed to fully break apart stool pellet. Stool suspension was centrifuged at 3,000 × *g* for 10 min at 4°C, and the supernatant was collected. An additional 10 mL of protease inhibitor cocktail tablet solution was added to the stool pellet, and the sample was vortexed for 5 min followed by another 3,000 × *g* for 10 min at 4°C. The supernatants (~25 mL) were pooled and centrifuged at 3,000 × *g* for 30 min. Pooled supernatants were filtered through fast-flow filter paper to remove the remaining stool debris. The flow through was centrifuged at 40,000 × *g* for 1.5 h to pellet the large EVs, and the supernatant was collected and filtered using 0.22 µm filter to remove microbes. To pellet the final small EVs, the 40,000 × *g* supernatant was ultracentrifuged at 167,000 × *g* for 2.5 h. This pellet was carefully washed twice with PBS and resuspended in ~50 µL PBS. EVs were freshly used or stored at −80°C until EV characterization and RNA isolation.

### RNA isolation and quantitative gene expression

Total RNA was extracted from stool EVs isolated by UC using the miRNeasy Mini Kit (Qiagen) per the manufacturer’s instructions. Purified RNA was eluted in 30 µL RNase-free water and stored at −80°C until use. RNA concentration was determined using a ND-1000 Spectrophotometer (Nanodrop Technologies). Fifty nanograms of total RNA isolated from the stool EVs was treated with DNase (ThermoFisher) and a standard reverse transcription using MultiScribe Reverse Transcriptase with random hexamers and stem loop primers (ThermoFisher) to specific mature miRNAs and U6 were used to generate cDNA. The thermocycling conditions are as follows: 16°C for 30 min, 42°C for 30 min, and 85°C for 5 min. cDNA was diluted 1:33 and qPCR was performed on a QuantStudio 7 Flex Real-Time PCR System (Applied Biosystems). Detection using SYBR Green to in-house designed qPCR primers (18S, 16S, FliC, and FliC K12) and Taqman Assays (mature miRNAs and U6) targets (ThermoFisher). Gene expression analysis is presented as mean CT values in triplicate. qPCR primer sequences are as follows: (i) 18S FP 5′ GTAACCCGTTGAACCCCATT 3′, RP 5′ CCATCCAATCGGTAGTAGCG 3′. (ii) 16S FP 5′ CCAGCAGCCGCGGTAATAC 3′, RP 5′ TCAGATGCAGTTCCCAGGTTG 3′. (iii) FliC FP 5′ GTGACGGTACAGCGTTTGATG 3′, RP 5′ AGCAGCAGAACCTGTTGTTACG 3′. (iv) FliC K12 FP 5′ TGGTGCTACCACCACAAACAA 3′, RP 5′ ATCACCACCGGTGATTTTCG 3′.

### Nanoparticle tracking analysis

The high-resolution particle size distribution and concentrations of EVs were determined using NTA by calculating light scattering and Brownian motion properties. Briefly, isolated EVs were diluted 500-fold using ion-free PBS and injected into a Nanosight NS300 instrument (Malvern Instruments, Westborough, MA, USA) equipped with a blue488 laser and a sCMOS camera. The samples were measured for 60  s and five movies were taken for each sample with the single shutter (1,300) and gain (512) mode at 25 FPS. The analysis settings were (i) threshold: 6; (ii) blur size: auto; and (iii) max jump distance: Auto: 13.1–17.1 pix; temperature: 23.6–23.8°C. For determining particle concentration, dPBS was run as a blank for background particle concentration.

### Cryo-transmission electron microscopy

Cryo-transmission electron microscopy (Cryo-TEM) from the University of Florida ICBR was used to image EVs. In brief, 3 µL of a suspension of concentrated mouse stool EVs was pipetted onto glow-discharged, C-Flat holey-carbon grids (Protochips Inc.). The grids were immediately vitrified in a slurry of liquid ethane cooled by liquid nitrogen in a Mark IV Vitrobot (FEI Co.) at 4°C and 95% humidity. The grids were examined, and images gathered on a FEI Tecnai G2 F20-TWIN microscope (RRID:SCR_019146) operated at 200 kV and ~20 e−/Å2 dosage (low dose). The scale bars are 100 nm.

### Library construction and miRNA sequencing of stool EV RNA

miRNA sequencing was performed on the RNA isolated from individual stool pellets of GF group, C13 group, and C13 + *C. jejuni* group 6 days after bacteria colonization. In brief, total RNA from EVs was isolated following the manufacturer’s protocol of miRNeasy Mini kit (Qiagen). Isolated RNA was run on Agilent 2100 BioAnalyzer to determine the amount of miRNA from the total small RNA concentration. One sample each from the GF and the C13 groups was excluded from miRNA library preparation and sequencing due to low miRNA concentration (less than 10 ng) as determined by the Bioanalyzer, resulting in a final sample size of GF (*n* = 8), C13 (*n* = 5), and C13 + *C. jejuni* (*n* = 8). The amount of RNA used in library preparation was standardized to 10 ng of miRNA for each sample. miRNA sequencing libraries were synthesized and gel size-selected using the NEBNext Multiplex Small RNA Library Prep Set for Illumina (NEB). Samples were sequenced by the University of Florida NextGen DNA Sequencing (NS) core (RRID:SCR_019152) core on the Illumina Novoseq 6000 instrument (2  ×  150 run, S4 Flow cell).

### miRNA analysis

Data were analyzed as described previously (15). Briefly, CAP-miRSeq (39) was used to process the miRNA sequences as follows: reads were filtered and trimmed using cutadapt (40). Identification and quantification of miRNA were done using miRDeep2 (41), and differential miRNAs analysis was detected using edgeR. We considered a miRNA differentially expressed if its edgeR *P*_adj_ -value < 0.05. PCA was created using R’s prcomp function from the normalized and log transform miRNA counts and differences in samples clustering were tested using gls (Generalized Least Squares model) function in R package nlme.

Bacterial target prediction for mouse miRNA was done using PITA (42); an miRNA target prediction tool that evaluates the thermodynamic free energy between the miRNA-target complex. We considered a bacterial gene a potential target for a particular mouse miRNA if its ΔΔG score < −10 kcal/mol. To analyze miRNA and mouse gene targets, multiMiR (http://multimir.org/) was used.

### Statistical tests

We used R’s two-sample Wilcoxon test (also known as Mann-Whitney test) and for comparisons involving more than two, we corrected the *P*-values for multiple testing using R’s p.adjust function with method set to BH (Benjamini and Hochberg) (43).

## Data Availability

miRNA sequencing reads have been deposited in the National Center for Biotechnology Information (NCBI) Sequence Read Archive (SRA) under accession number PRJNA1209041.

## SUPPLEMENTAL MATERIAL

Table S1 spectrum.03184-25-s0001.xlsx Percentage of miRNA in total small RNA extracted from Fecal EVs in Germ-free, C13, and C13 + *C. jejuni* mice.

Table S2 spectrum.03184-25-s0002.xlsx Differentially expressed host EV-derived miRNAs in Germ-free, C13, and C13 + *C. jejuni* mice.

Table S3 spectrum.03184-25-s0003.xlsx Predicted miRNAs, their mouse gene transcripts, and RNA expression levels.

Table S4 spectrum.03184-25-s0004.xlsx miRNAs predicted using PITA to target bacterial gene transcripts.

Table S5 spectrum.03184-25-s0005.xlsx miRNAs predicted using PITA to target colibactin and cytolethal distending toxin gene transcripts.

Table S6 spectrum.03184-25-s0006.xlsx Predicted miRNAs, their mouse gene transcripts, derived from colon spatial transcriptomics data.

ASM does not own the copyrights to Supplemental Material that may be linked to, or accessed through, an article. The authors have granted ASM a non-exclusive, world-wide license to publish the Supplemental Material files. Please contact the corresponding author directly for reuse.
